# Flagella and Swimming Behavior of Marine Magnetotactic Bacteria

**DOI:** 10.3390/biom10030460

**Published:** 2020-03-16

**Authors:** Wei-Jia Zhang, Long-Fei Wu

**Affiliations:** 1 Laboratory of Deep-Sea Microbial Cell Biology, Institute of Deep-sea Science and Engineering, Chinese Academy of Sciences, Sanya 572000, China; wzhang@idsse.ac.cn; 2 International Associated Laboratory of Evolution and Development of Magnetotactic Multicellular Organisms, F-13402 CNRS-Marseille, France/CAS-Sanya 572000, China; 3 Aix Marseille Univ, CNRS, LCB, IMM, IM2B, CENTURI, F-13402 Marseille, France

**Keywords:** flagellar number and position, north-seeking and south-seeking, magnetic and photo-response

## Abstract

Marine environments are generally characterized by low bulk concentrations of nutrients that are susceptible to steady or intermittent motion driven by currents and local turbulence. Marine bacteria have therefore developed strategies, such as very fast-swimming and the exploitation of multiple directional sensing–response systems in order to efficiently migrate towards favorable places in nutrient gradients. The magnetotactic bacteria (MTB) even utilize Earth’s magnetic field to facilitate downward swimming into the oxic–anoxic interface, which is the most favorable place for their persistence and proliferation, in chemically stratified sediments or water columns. To ensure the desired flagella-propelled motility, marine MTBs have evolved an exquisite flagellar apparatus, and an extremely high number (tens of thousands) of flagella can be found on a single entity, displaying a complex polar, axial, bounce, and photosensitive magnetotactic behavior. In this review, we describe gene clusters, the flagellar apparatus architecture, and the swimming behavior of marine unicellular and multicellular magnetotactic bacteria. The physiological significance and mechanisms that govern these motions are discussed.

## 1. Introduction

**Figure 1:**
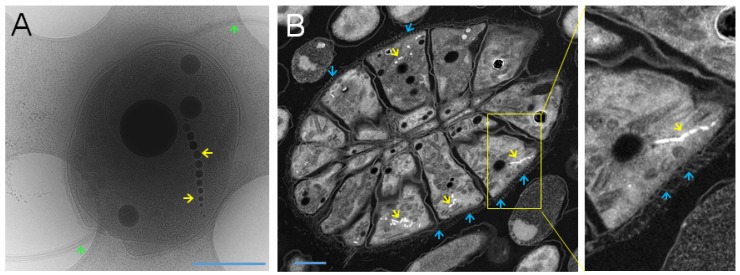
Magnetosomes and flagella of magnetotactic bacteria. (**A**) Bilophotrichously flagellated MO-1 cells possess two sheathed flagellar bundles (green arrow) and one magnetosome chain (yellow arrow). (**B**) Peritrichously flagellated ellipsoidal magnetoglobule with flagella (blue arrows) and magnetosomes (yellow arrows). Only the portion of flagellar filaments in the surface matrix was preserved during sample preparation. Scale bar is equal to 0.5 µm. Courtesy of the electron cryotomography micrograph (**A**) from Dr. J. Ruan and Professor K. Namba, and of the Scan-TEM high-angle annular dark-field (STEM-HAADF) mode micrograph (**B**) of ultrathin sections of high-pressure freezing/freeze substitution fixation (HPF/FS) fixed ellipsoidal magnetoglobule from Professor N. Menguy and Dr. A. Kosta.

Magnetotactic bacteria (MTB) are a group of phylogenetically, morphologically, and physiologically diverse Gram-negative bacteria [1,2]. They share the common capability of synthesizing unique intracellular organelles, the magnetosomes, i.e., single-domain magnetic crystals of magnetite or greigite, which are enveloped by membranes (Figure 1). Cytoskeleton MamK filaments enable the magnetosomes to be organized into chains [3,4,5]. Magnetosome chains impart a net magnetic dipole moment to the cell, which allows cells to align and swim along geomagnetic field lines [6]. This behavior, referred to as magnetotaxis, is believed to facilitate microaerophilic or anaerobic MTB to locate at the preferable oxic–anoxic interface in chemically stratified sediments or water columns [1].

Phylogenetically, magnetotactic bacteria are members of several classes of the Proteobacteria phylum including the Alpha-, Gamma-, Delta-, Zeta-, *Candidatus* Lambda-, *Candidatus* Eta-classes, the Nitrospirae phylum, the *Candidatus* Omnitrophica phylum, the *Candidatus* Latescibacteria phylum, and the Planctomycetes phylum [7]. They present various morphotypes including cocci, spirilla, rod-shaped, vibrio, and more complex multicellular magnetotactic prokaryotes that are also called magnetoglobules (MMP) [1,8].

Magnetotactic bacteria are found worldwide in aquatic environments from freshwater to marine ecosystems. Here, we will discuss mainly three types of marine magnetotactic bacteria because of their complex flagellar architecture and peculiar motile behavior. The first is the spirillum *Magnetospira* sp. strain QH-2 isolated from the intertidal sediments of the China Sea [9]. Phylogenetically, QH-2 belongs to Rhodospirillaceae and is closely related to two freshwater magnetotactic spirilla, *Magnetospirillum magneticum* AMB-1 and *Magnetospirillum gryphiswaldense* MSR-1. Yet, certain traits such as the synthesis of osmoprotectant, Na^+^-dependent NADH-quinone oxidoreductase, and Na^+^-motive force driven flagellar motors, make QH-2 better suited to a marine sedimentary lifestyle than its freshwater counterparts [10]. The second is the ovoid-coccoid *Magnetococcus massalia* strain MO-1 isolated from sediments of the Mediterranean Sea (Figure 1A) [11]. MO-1 belongs to the newly established class *Candidatus* Etatproteobacteria and possesses the most exquisite flagellar apparatus [12]. The third group is the magnetoglobules that have developed both multicellular and magnetotactic properties during their evolution. To date, magnetotactic multicellular prokaryotes are found only in marine environments [8]. They exhibit peculiar patterns of motility by coordinatively rotating tens of thousands of peritrichous flagella (Figure 1B), including both polar and axis magneto-aerotaxis, ping-pong motion, and photophobic and photokinesis swimming patterns. 

## 2. Flagellar Apparatus of Marine Magnetotactic Bacteria 

Flagella provide one of the most highly efficient means of bacterial locomotion and play a pivotal role in adhesion, biofilm formation, and host invasion [13,14,15,16]. Bacterial flagella share a basic tripartite structure; the basal body, the hook, and the filament [17]. The basal body contains a reversible rotary motor made of a rotor, a drive shaft, a bushing, and about a dozen stators. The stator forms the proton or sodium ion pathway and converts ion flow across the cytoplasmic membrane into the mechanical work required for flagellar motor rotation. The basal body also contains the flagellar protein export apparatus, which recognizes, unfolds, and translocates flagellar components into the central channel and to the distal, growing end of the flagellum [15,17]. Flagellar filaments have a helical structure and function as a screw, where rotation pushes or pulls the cell. Despite structural similarities, bacterial flagella exhibit extensive variations in both number and placement between species, and this criterion had been used in bacterial taxonomy in the past. Bacteria may have a single flagellum (monotrichous) at one end of the cell (polar flagellum), or a single flagellum at both ends (amphitrichous), numerous flagella in a tuft (lophotrichous), or flagella distributed all over the cell (peritrichous). The three model magnetotactic bacteria reviewed here possess amphitrichous, bilophotrichous, and peritrichous flagella that underpin complex magnetotactic motion.

### 2.1. Flagellar Apparatus of Amphitrichously Flagellated Magnetospira sp. Strain QH-2

**Figure 2:**
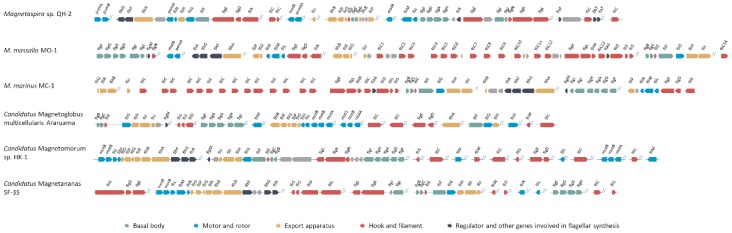
Organization of flagella genes in model magnetotactic bacteria. The data are derived from genomic data of amphitrichously flagellated *Magnetospira* sp. QH-2 [10], bilophotrichously flagellated *M. massalia* strain MO-1 [18] and *M. marinus* strain MC-1 [19], peritrichous flagellated spherical magnetoglobules Ca. M. multicellularis Araruama [20], Ca. Magnetomorum strain HK-1 [21], and ellipsoidal magnetoglobules Ca. Magnetananas updated from the incomplete genome sequence [22]. Separated localization of the gene clusters is marked by double slashes. Arrows show the genes and their transcriptional direction; their lengths are proportional to the size of the genes.

The spirillum *Magnetospira* sp. strain QH-2 was isolated from the intertidal sediments of the China Sea [9]. The cells are amphitrichously flagellated with a single flagellum at each pole, their composition and structure are probably the simplest when compared to the bilophotrichous flagella of MO-1 and the peritrichous flagella of the multicellular magnetoglobules. Genomic analysis identified flagellum synthesis genes at 10 locations (Figure 2) [10]. Intriguingly, multiple genes coding for either proton-driven or sodium ion-driven motors were identified, including a single *pomA*, a single *motB*, and two complete sets (*pomAB*/*pomA*-*motB*), although the paralogs share limited similarity (below 45%). In addition to the flagellar biosynthesis genes, well conserved in most prokaryotes, two genes annotated as O-b-N-acetylglucosaminyltransferase were identified in flagellar gene clusters. They contain a flagellin and several flagellar biosynthesis regulatory genes, demonstrating their function in flagellin glycosylation. 

### 2.2. Flagellar Apparatus of Bilophotrichously Flagellated M. Massalia Strain MO-1

*M. massalia* strain MO-1 synthesizes two sheathed flagellar bundles on the long axis side of its ovoid body (Figure 1A). Each bundle is composed of 7 flagella and 24 fibrils. The flagella are organized in a 2:3:2 array, and each of them is surrounded by 6 fibrils; altogether they constitute seven intertwined hexagonal arrays [12]. It has been hypothesized that the 24 fibrils might counter rotate between the 7 flagellar filaments to minimize the friction that would be generated if the flagella were directly packed together in a tight bundle [12]. The closely related *M. marinus* strain MC-1 and several marine bilophotrichously flagellated magnetotactic cocci seem to possess a flagellar apparatus with a similar architecture [11,23,24,25,26]. Recently, an even more complex flagellar apparatus consisting of 19 flagella arranged in a 3:4:5:4:3 array within the flagellar bundle has been observed in a magnetotactic cocci found in the biogenic sediments of a Mariana–Yap seamount [27]. Questions inevitably arose about these exquisite flagellar apparatuses, such as: What is the factor that determines the accurate localization of flagella and why are they constrained within a sheath structure?

Sheath or pseudo-sheaths, each enclosing a single flagellum, have been reported for *Caulobacter crescentus* [28], *Pseudomonas rhodos* [29], *Vibrio* spp. [16], *Helicobacter pylori* [30], and *Bdellovibrio bacteriovorus* [31]. These flagellar sheath structures are believed to be an extension of the outer membrane. In contrast, the sheath of MO-1 is assembled from a large (>350 kDa) glycoprotein and in a calcium ion-dependent manner made into a left-handed helical structure [12,32]. The sheathed bundle of seven flagella produces a thrust force, which is nine times greater than an unsheathed one, and this is indispensable for the smooth swimming motion of MO-1 cells [12,32]. In addition, as all strains possessing similar flagellar structures reside in marine sediments, the presence of a sheath could possibly protect the filaments from breaking whilst moving through sands, and this implies that there is an evolutionary adaptation to this habitat. 

The complexity of this bilophotrichous sheathed flagellar is further demonstrated by using genome sequences. Genomic analysis revealed that the genetic structures of flagellar synthesis genes in strains MO-1 and MC-1 are well conserved (Figure 2). Most intriguingly, they possess the highest number of flagellin paralogs (14 flagellin genes in strain MO-1 and 15 in strain MC-1) found in bacterial genomes to date [19,33]. In both strains, most flagellin genes are spread in a tandem array, while a single *fliC* resides in a more compact flagellar gene cluster consisting of *flgKL* (hook-associated proteins), *fliW* (antagonist of general regulator CsrA), a putative *flaG* gene (function unknown), *fliD* (filament cap), and two *fliS* (chaperon) (Figure 2). There is no obvious element, such as insertion-sequence (IS) elements or duplicated flanking sequences, which could explain the mechanism of duplication of these *fliC* paralogs. As indicated by quantitative PCR (q-PCR) and mass spectrometry analyses, all 14 flagellins in MO-1 are expressed, highly glycosylated, and present in the flagellar filaments, although they differ significantly in quantity [33]. The biological significance of highly redundant flagellins and the way they make up the filament, i.e., whether each flagellin forms an individual simple filament or whether multiple flagellins form complex segmented or mosaic filaments, requires in-depth research. Nevertheless, the flagella of MO-1 cells show unprecedented complexity in spatial organization and flagellin redundancy in unicellular microorganisms. 

### 2.3. Peritrichous Flagella of Multicellular Magnetoglobules

There are two kinds of magnetoglobules. In 1983, Farina et al. discovered spherical or mulberry-like magnetoglobules in the Rodrigo de Freitas lagoon in Brazil [34]. Typically, 15–45 bacterial cells arrange themselves with a helical geometry in a multicellular entity [35]. Since then, these types of magnetoglobules have been observed worldwide [36,37,38,39,40,41,42]. The second morphotype, the ellipsoidal or pineapple-like magnetoglobules, were observed in the Mediterranean Sea [8,43,44], the China Sea, and the Pacific Ocean [45,46,47,48,49]. Approximately 60 cells axisymmetrically assemble along the longitudinal axis to achieve a one-layer hollow entity that is held by a lattice at the surface [8]. Phylogenetic studies have identified eleven species belonging to six genera of spherical magnetoglobules and nine species belonging to six genera of ellipsoidal magnetoglobules. They formed branches of a magnetoglobule clade, which are affiliated with Deltaproteobacteria, but are distinguished from another multicellular Deltaproteobacteria, the myxobacteria [8]. Both morphotypes exhibit a conspicuous periphery–core architecture. Juxtaposed membranes adhere together cells surrounding the core lumen where material and information exchange may occur among the cells. Magnetoglobules possess multiple magnetosome chains arranged along their long axis at the cell periphery. The surface of magnetoglobules is covered by approximately tens of thousands of flagella (Figure 1) [8,50]. 

Genomic analysis revealed the following salient features of the genes required for flagella synthesis in magnetoglobules. First, they possess well-conserved gene clusters containing (*motA*)-2*motB*-*fliRQPONL*-*flhB*-(*flhAFG*-*fliA*) (Figure 2). Second, they have multiple copies of several genes involved in motor rotation, such as *motAB* that code for proton–ion driven motors and *fliN* codes for a part of the motor switch complex, which modulates the motor activity. It is noticeable that the second copy of *fliN* is twice the size of the copy in the conserved cluster. The long *fliN* of the switch complex component might be involved in the coordination of flagellar rotation. Third, they have 2–3 copies of flagellin *fliC* genes, of which one copy is longer than the usual *fliC* genes. Finally, the *flhF* and *flhG* genes controlling the flagellar number and position are highly conserved in magnetoglobules. They may bear intrinsic characteristics for the regular implantation of thousands of flagella at the outer surface of magnetoglobule cells.

## 3. Magnetotaxis Behavior of Marine Magnetotactic Bacteria

Magnetotactic bacteria are capable of aligning and swimming along the geomagnetic field lines. The efficiency of magnetic orientation depends on the local redox gradient and latitude of the habitats where the MTB dwell, as well as on the flagellar apparatus of MTB cells.

### 3.1. Polar and Axial Magnetotaxis

Magnetotaxis and aerotaxis work together in MTB to perform a so-called “magneto-aerotaxis”. Two different magneto-aerotactic mechanisms, termed polar and axial magnetotaxis, are found in different bacterial species [1,24]. In droplets of samples on a microscope slide or cover, there is an oxygen gradient that is created due to the diffusion of oxygen from the peripheric edge toward the center. When inspected with the optical microscope under oxic conditions, polar magnetotactic bacteria swim persistently in one direction, either the north or the south, in the magnetic field. In contrast, axial magnetotactic cells swim in either direction along the magnetic field lines with frequent, spontaneous reversals of swimming direction without turning around. 

The bilophotrichously flagellated *M. massalia* strain MO-1 exhibits a polar magnetotactic behavior, swimming northwards along the geomagnetic field lines by means of two sheathed flagellar bundles, at speeds of up to 300 µm/s, with frequent changes from a right to a left hand helical trajectory [11]. Freshwater amphitrichously flagellated *M. magneticum* AMB-1 shares a similar morphology with the marine *Magnetospira* sp. strain QH-2, and its swimming behavior has been the most extensively studied. Asymmetric rotation of the flagella (counterclockwise at the lagging pole and clockwise at the leading pole) enables the cell to “run” while symmetric rotation triggers cell tumbling [51]. AMB-1 cells frequently tumble and change swimming direction, displaying the typical axial magnetotactic behavior. Peritrichous magnetoglobules collected from the Mediterranean Sea swim preferentially northward, a polar magnetotaxis. However, at times, some of them randomly change swimming direction southward and subsequently change back to a north-seeking swim [8]. This is a typical behavior of axial magnetotaxis. The stochastic backward motion may play a similar physiological function to the tumbling of *Escherichia coli* that allows bacteria to randomly explore the favorable direction in which to go. Therefore, a given MTB may perform both polar and axial magnetotactic motilities that are not reciprocally exclusive, and the alternative usage is part of the adaptation strategy.

### 3.2. Bounce Motion

**Figure 3:**
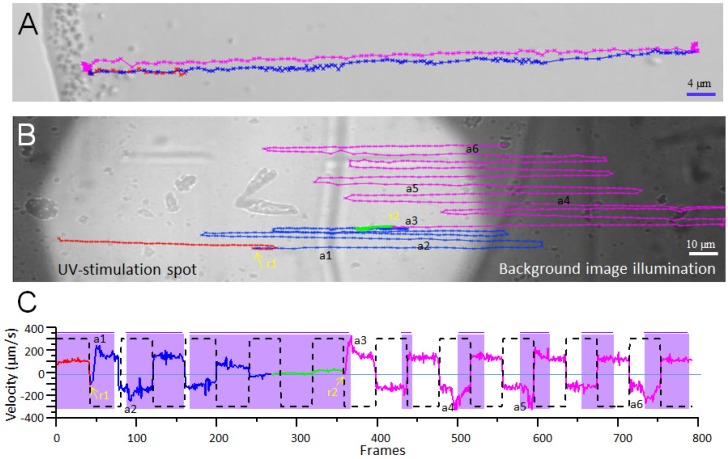
Ping-pong motion and photo-sensitive motility. (**A**) a magnetotactic bacillus of ~ 4 µm swims northward (red track) until the edge of the droplet. Then, it swims southward, opposite to the north-seeking swimming direction to the center of the droplet (blue track), which is followed by a returning north-seeking (magenta track). (**B**) is a representative photosensitive swimming behavior of magnetoglobules and (**C**) is an ImageJ analysis of the data [8]. The dot-line square curve indicates the north direction of the alternating magnetic field. Positive velocity means that the magnetoglobule swims from left to right on the image whilst the negative values are opposite. The velocity curve colors in (**C**) correspond to the same colors of the swim tracks in (**B**). When the velocity curve is on the same side as the field curve using zero velocity line as a reference, the magnetoglobule exhibits a north-seeking magnetotaxis (e.g., red and green tracks), otherwise it displays a south-seeking magnetotaxis (blue and magenta tracks). Violet areas show the swimming of the magnetoglobule in the UV spot. Yellow arrows with r1 and r2 indicate the sudden change of swimming direction to south-seeking; a1 to a6 show the accelerations.

Magnetoglobules display a canonical escape or ping-pong motion. It is composed of a sudden accelerated excursion from the droplet edge towards the center opposing the direction of magnetotaxis. At variable distances, they decelerate, stop, and swim with acceleration back to the droplet edge [8,34,35,36,41,42,44,45,48,52,53,54,55,56,57]. In fact, the ping-pong motion is not restricted to magnetoglobules; other morphotypes of MTB also display this kind of motility. The small cell sizes make observations difficult. Some of the big rod-shaped MTB exhibit obvious escape motion as shown in Figure 3A and Vdieo S1 ping-pong motion of big rod-shaped MTB. 

The ping-pong motion can be observed when cells hit the edge of the droplets or other kinds of obstacles, such as the wall of microchannels [8]. In addition, both the unicellular *M. massalia* strain MO-1 [58] and multicellular magnetoglobules [8] exhibit a conspicuous backward motion when they encounter particles. In all conditions, cells are prevented from swimming in a magnetotaxis direction, and exhibit a bounce motion. The mechanism involved in the mechanical sensing of microchannel walls and particles might be different from that of the surface/border of the droplets.

### 3.3. Photo-Sensitive Magnetotaxis: Photophobic Response and Photokinesis

Sunlight consists of electromagnetic waves, of which high energetic radiation is harmful for living organisms. Fortunately, the geomagnetic field protects living beings from the deleterious effect of radiation. In addition, the geomagnetic field provides a pervasive and reliable source of directional and positional information for various organisms to use as an orientation cue, which maps migrating or homing routes. Magnetotactic bacteria have developed means of sensing not only the geomagnetic field, but also certain wavelengths of sunlight.

Microbes react to light illumination in different ways depending on their physiological properties. Phototaxis refers to cells swimming along the direction of a light beam towards (positive) or away from (negative) a light source [59]. In reaction to a sudden change of light intensity, photophobic microbes will swim to lower intensity whereas scotophobic microbes will move to higher intensity regions. Photokinesis describes the change in velocity (speed and direction) in response to light. The freshwater *M. magneticum* AMB-1 exhibits phototaxis behavior that is independent of the wavelength and magnetotaxis [60]. Photophobic swimming has been reported for unicellular *Magnetospira* sp. QH-2 [9] and multicellular magnetoglobules [20,37,41,42,44,45,48]. In this case, magnetotaxis drives cells to the edge of droplets. In reaction to illumination with blue (450-480 nm), violet (400-410 nm), and ultraviolet light (330-385 nm), the bacteria swim towards the center, in the opposite direction of magnetotaxis, with increased acceleration, which is similar to ping-pong motion, but is distinct due to the absence of a return swim. The reaction time is proportional to the wavelength: the shorter the wavelength, the quicker the reaction. 

Interestingly, ellipsoidal magnetoglobules show a photokinesis behavior. Reverse fluorescence microscopes generally have two light-sources. One is a transmission background visible light (tungsten halogen lamp) for observation and imaging, and the other is an epi-illumination for fluorescence excitation. The second light source can be used to analyze the photo effect on swimming behavior by illumination at a given wavelength on a defined area [8]. The swimming of ellipsoidal magnetoglobules collected from the Mediterranean Sea was maintained within the illumination spots via the application of an alternate uniform magnetic field in order to periodically reverse the swimming direction of magnetoglobules (Figure 3B). At times, magnetoglobules suddenly changed their swimming direction being opposite to the initial magnetotaxis direction with increased acceleration, when stimulated with UV light (385 nm). Variable proportions of magnetoglobules reacted to violet (430 nm) [8]. This is a typical photokinesis behavior, i.e., changing the swim speed and direction. The dependence of the wavelength and intensity of the light stimulus remains to be characterized.

### 3.4. Physiological Function of Magnetotaxis

Magnetotactic bacteria live at, or just below, the oxic–anoxic interface or redoxocline in aquatic habitats. Interestingly, magnetotactic bacteria collected from the Northern Hemisphere swim preferentially northward, in parallel with the geomagnetic field lines (north-seeking (NS)) [23], and those from the Southern Hemisphere swim preferentially antiparallel to the geomagnetic field lines to the magnetic south pole (south-seeking (SS)) [61]. The geomagnetic field is inclined downward from horizontal in the Northern Hemisphere, and upward in the Southern Hemisphere, with the inclination magnitude increasing from the equator to the poles. Therefore, the hypothetical physiological function of magnetotaxis can be that magnetotaxis guides the cells in each hemisphere downward to the less oxygenated regions of the aquatic habitat [1]. 

Marine sediments are characterized by opposing oxygen and reductant (e.g., sulfide) gradients within the upper millimeters of the sediments, which are covered by air-saturated seawater. The pattern of the gradients constantly changes due to the convective water currents at the sediment surface, dynamic metabolism of microbe populations, or periodic exposure to the air during low tide. In order to adapt to these ever-changing environmental parameters, magnetotactic bacteria have to combine magnetotaxis with aerotaxis. Moreover, penetration downward from the water phase into the sediments and swimming in the water pockets requires robust flagellar propellers. As a consequence of environmental selection, the *M. massalia* strain MO-1 synthesizes sheath-protected, well-organized, and highly coordinated flagellar apparatus that ensure a high swimming velocity [11]. When they encounter the bulk of an obstacle, MO-1 cells can squeeze through them or change direction using the bounce motion, thereby circumventing the obstacles [58]. Backward swimming occurs using various angles between the translation and field axes, which provides a large range of swimming directions in order to circumvent the obstacle. The robust flagellar apparatus and versatile swimming capacity give MO-1 cells a competitive fitness in marine sediments. In addition, studies with the axenic culture of MO-1 provide compelling evidence to support the physiological significance of magnetotaxis. 

The cultures in polystyrene plastic tubes exhibit a vertical downward oxidation–reduction potential (ORP, or redox) gradient, and a radiate gradient with an oxygen concentration decrease from the peripheral zone to the center, due to the diffusion of oxygen across the tube wall. During growth, MO-1 generates an oxic–anoxic–oxic oxycline pattern and forms two bacterial swarm bands. Remarkably, the upper band, where the magnetic field is parallel to the direction of the redox potential decrease, consists of >95% north-seeking (NS) cells, while the lower bacterial band, where the downward magnetic field lines are opposite to the upward direction of redox potential decrease, is composed of >90% south-seeking (SS) cells [62]. In both loci, cells with the ‘correct’ magnetotaxis polarity that are directed to swim towards the direction of the redox potential decrease are selected. Therefore, these observations are consistent with the hypothesis of magnetotaxis function and indicate the configuration of the ORP and magnetic field direction on a magnetotactic direction [62]. Further analysis by incubating MO-1 cells in a shielded, hypo-magnetic field (2 nT) showed that bacterial growth produces irregular forms of oxycline. Most importantly, the biomass of the cultures incubated in a hypo-magnetic environment are two orders of magnitude lower than those in the geomagnetic field, and could not grow at all when inoculated with a low quantity of cells [58]. This clearly demonstrates that magnetotaxis does present an advantage for the growth of MO-1 in the oxycline, and is even essential at low cell densities.

Magnetoglobules swim faster than most unicellular bacteria and are large in size, which has an advantage in mitigating the risk of predation. Magnetoglobules dwell in intertidal sediments as deep as 30 cm and undergo seasonal vertical movement in response to nutrient distribution changes [63]. Shapiro et al. have suggested that photophobic behavior enables magnetoglobules to optimize their location to adapt to circadian variations in chemical gradients and light intensity [37]. Indeed, genes involved in controlling the circadian rhythm have been found in the genomes of magnetoglobules [20,22]. Therefore, magnetoglobules seem to have adopted a multicellularity and photosensitive magnetotaxis in order to adapt to shallow marine environments. 

## 4. Mechanism of Magnetotaxis

In seeking an environment optimal for their growth, bacteria change swimming direction frequently by changing the direction of flagellar rotation. Our current understanding of chemotaxis stems mainly, from the extensively studied, peritrichously flagellated enterobacteria *E. coli* and *Salmonella* spp. [15]. These alternate between periods of “run” and “tumble” and the swimming pattern is determined by the direction of the flagellar motor rotation. When the motor rotates in the counterclockwise (CCW) direction (as viewed from the distal end of the filament), several flagellar filaments form a loose bundle to propel the cell forward to run. When the motor reverses its rotation to clockwise (CW), the bundle falls apart and the cell tumbles [15]. Monopolar flagellum pushes marine vibrio forward by CCW rotation and pulls it backwards through CW rotation [64]. According to the prevailing hypothesis, magnetotactic bacteria align passively along the geomagnetic field lines, which guide swimming downward from the oxic zone to the oxic–anoxic interface, by rotating their flagella counterclockwise [1]. When located in the anoxic zone magnetotactic bacteria swim upward by reversing the direction of flagellar rotation from counterclockwise to clockwise. This is a simplified assumption, because it does not explain the coordinated rotation of bilophotrichous flagella of MO-1 and tens of thousands peritrichous flagella of magnetoglobules. MO-1 cells swim for very long distances without stopping, until they encounter an obstacle, which causes them to turn their bodies and swim against the magnetic field to circumvent the obstacle [58]. Such behavior is in contrast with the model of aligned forward–backward motion. The ovoid MO-1 cells possess two flagellar bundles on the long axis side of their body (Figure 1) [11,12]. These cells rotate around and translate along their short body axis [58]. It is consistent with the fact that the two flagellar bundles are placed on the long body axis, thus presumably generating the propulsion along the short body axis [65]. Electron cryotomography (ECT) analysis revealed that the magnetosome chains in MO-1 cells are roughly along the long body axis in >90% of cells, or with angles of less than 45° to the short body axis in 5% of cells [12]. Therefore, the direction of the magnetic moment is not parallel to the short body axis in most MO-1 cells, hence MO-1 cells are not perfectly aligned along the magnetic field lines while they swim. The direction of magnetic dipole moment exhibits a cyclical change perpendicular to their translation direction. The poor alignment of magnetic moment along the magnetic field lines enables backward swimming with a body reversal in bounce motion. In contrast, ellipsoidal magnetoglobules align well in magnetic fields and their bodies remain in the same direction when swimming backwards [8]. It is noteworthy that the backward swimming in bounce motion and the axial magnetotaxis of magnetoglobules start with the highest acceleration and have higher instantaneous velocity than the forward swimming. Hence, magnetoglobules seem to steer their flagella according to the magnetic direction of their swimming. Greenberg et al. have analyzed the kinematics of ping-pong motility in magnetic fields and proposed a receptor-mediated mechanism for sensing the magnetic field by spherical magnetoglobules [56]. 

Bacteria can sense a wide range of environmental signals that steer bacterial locomotion through the extensively studied chemotaxis mechanism [64,66]. The chemoreceptors, methyl-accepting chemotaxis proteins (MCPs), detect the stimuli, and control, through histidine protein kinase CheA, the phosphorylation state of the response regulator CheY. Phospho-CheY interacts with the flagellar motor and switches the rotation direction. Rotation in one direction results in smooth swimming, whilst switching the rotation direction may lead to backward motion, tumbling, or stopping swimming, depending on the bacterial species [64]. We have proposed a chemotaxis-like magnetotaxis mechanism for the freshwater *M. magneticum* strain AMB-1. We have shown that the Amb0994, an MCP-like protein, lacks the periplasmic signal molecule-binding domain, and interacts with cytoskeleton MamK filaments, on which the magnetosome chain is connected [67]. Our hypothesis is that poor alignment of magnetosome chains in the magnetic field would generate a magnetic torque that applies a mechanical strength on the MamK filament. Interaction between the MamK filament and Amb0994 converts the mechanical signal to a biochemical signal, i.e., phosphorylation of CheA. Subsequent phosphorylation of CheY and its binding onto the flagellar motor would slow down or stop the rotation of flagella, to avoid them from swimming in the wrong direction. Two results are consistent with this hypothesis. Overexpression of Amb0994 interferes with the AMB-1 response to the reversal of the magnetic field [67]. Deletion of the *amb0994* gene resulted in the failure of AMB-1 cells to align with the magnetic field lines in a weak biologically relevant magnetic field, and this dysfunction was recovered by in trans complementation of the mutant [68]. These results support the chemotaxis-like magnetotaxis mechanism. Considering the morphological and physiological diversity of magnetotactic bacteria, various magnetotactic mechanisms might be used.

Coordinated swimming behavior is a fundamental feature that emerged during the evolution of multicellularity. Magnetoglobules exhibit a highly complex motion: polar and axial magnetotaxis, bounce motion, photophobic response, and magneto-photokinesis [8]. Bacterial photo-sensing might rely directly on dedicated photoreceptors, or indirectly on the products of photosynthesis or other illumination by-products, i.e., reactive oxygen species, ATP, change of intracellular redox, or force proton motif. Six types of photosensory proteins using four kinds of chromophores are well characterized [69,70]. Among them, two groups, cryptochromes and sensory rhodopsins, are involved in photo-responsive motion.

Flavin-based cryptochrome serves as magnetoreceptor for migratory birds to exploit the geomagnetic field for direction and mapping [71]. Blue-light excitation of cryptochrome proteins in the retina creates a radical–pair consisting of molecules with a single unpaired electron. The spins of the two unpaired electrons are either antiparallel to one another (singlet state) or parallel (triplet state). As with a compass, the spin of one unpaired electron is primarily influenced by the magnetism of a nearby atomic nucleus, and the other is further away from the nucleus and influenced only by Earth’s magnetic field [71,72]. The difference in the field shifts the radical pair between two quantum states with differing chemical reactivity. Therefore, a change in surrounding magnetic field affects the interconversion and the reaction direction, which results in an output signal being transferred to the neural system in animals [71,73,74]. The radical pair compass is light-dependent, involves quantum entanglement, and is thus considered as a representative example of quantum biology [72]. 

Prokaryotic rhodopsins (proteorhodopsins) are involved in photomotility at two levels [75]. They function as photo-driven ion pumps, where proteorhodopsins translocate ions across cytoplasmic membrane and establish ion gradients upon capture of light. In turn, the gradients drive the flagellar motors for motility [76]. Sensory rhodopsins are directly involved in phototaxis of archaea *Halobacterium halobium,* which are attracted to long wavelength visible light (red-light attraction), and repelled by shorter wavelength light (blue-light repellence). Together, two phototaxis receptors, sensory rhodopsin I (SRI) and sensory rhodopsin II (SRII), and two transducers, haloarchaeal transducer for SRI (HtrI) and haloarchaeal transducer for SRII (HtrII) form two phototaxis reception complexes [77]. Retinal-containing SRI or SRII are transmembrane proteins that encircle cognate HtrI or HtrII. The transducers HtrI and HtrII are structurally and functionally similar to MCP proteins. The SR-Htr complexes modulate the CheA kinase activity and steer flagellar rotation through integration with a switch regulator CheY. Orange-light activates SRI that interacts with HtrI and transiently inhibits CheA kinase activity. Reduced concentrations of phosphorylated CheY decreases the probability of switching motor rotation. As a consequence, the cell continues swimming towards the orange light, displaying the red-light attraction behavior [77]. In contrast, blue-light activation of SRII excites transient activation of CheA, an increase of phospho-CheY concentration, and has the probability of switching flagellar motor rotation direction, which leads to the blue-repellence. In addition to these two simple, direct reaction processes, sequential activation of SRI by orange followed by near-UV results in a strong repellent response. Sensory proteorhodopsin has been found in marine bacteria but their physiological function has not been demonstrated yet [77].

We have not identified genes that encode for either cryptochrome or proteorhodopsin in the genomes of *Mangetospira* sp. QH-2 [10], *M. massalia* MO-1 [18], spherical magnetoglobules [21,78], or incomplete genomes of ellipsoidal magnetoglobules. Therefore, the photo-sensing observed in these magnetotactic bacteria might be performed with other kinds of photoreceptors, or indirectly through chemical and physical reactions. Short wavelength light induces photoreaction and creates active oxygen species, which modify physiological conditions and triggers cellular reaction. In ellipsoidal magnetoglobules, we observed the fence-like structure, which looks like photosynthetic membrane lamellae and could be an appropriate candidate for accommodating the photoreceptors involved in photo-sensing [8]. It might also function as a grating to relay and convert light signals. Multicellular magnetotactic prokaryotes displayed a helical trajectory of swimming and reacted to illumination with UV-light perpendicular to the translation direction. They changed the magnetotaxis direction and velocity suddenly within the illumination area. Therefore, the magneto-photokinesis is unlikely to be a result of the detection of an intracellular spatial light gradient. The sudden change of swim direction under constant illumination would suggest the cumulating effect of periodical exposure of photoreceptive structures to UV-light, or the production of harmful by-products. Therefore, multicellular magnetotactic prokaryotes reversed their swimming direction to escape from the deleterious light. It remains an enigma how thousands of flagella of 60–80 cells coordinate their rotations to propel the swimming direction away from the default magnetotaxis orientation. 

## 5. Conclusions

Magnetotaxis is an obvious magnetic field reactive swimming behavior, and little is known about the mechanism of magnetoreception. Despite extensive studies of magnetotactic bacteria over the last two decades, it remains a question of debate whether bacteria steer their flagellar motors in response to the state of their alignment in magnetic fields. Light is electromagnetic radiation and it affects magnetotaxis. What might be the connection between magnetic and optical stimuli? Photoreceptors known to be responsible for photomotion have not been identified in magnetotactic bacteria, in spite of the advances in metagenomics. The scarcity of axenic marine bacterial cultures makes the study of photo-sensitive magnetotaxis mechanisms even more complicated. The paradigm of bacterial chemotaxis is underpinned by intracellular diffusion of phosphorylated proteins and their binding to flagellar motors, in order to steer the swimming behavior in response to environmental stimuli [66]. How is a signal transmitted across multiple membranes to reach tens of thousands of flagellar motors at the surface of approximately 60 cells? Considering the particle and wave duality of photons, the application of the quantum concept might provide a solution and shed some light onto the complex study of magnetic photokinesis.

## Supplementary Materials

The following are available online at https://www.mdpi.com/2218-273X/10/3/460/s1, Video S1: ping-pong motion of big rod-shaped MTB.
